# Supplementary material for: Variants in the WDR44 WD40-repeat domain cause a spectrum of ciliopathy by impairing ciliogenesis initiation
Source: Nat Commun. 2024 Jan 8;15:365. doi: 10.1038/s41467-023-44611-2 (PMC10774338; doi:10.1038/s41467-023-44611-2)
Supplement: Supplementary file 1 — Supplementary Information [file 41467_2023_44611_MOESM1_ESM.pdf]

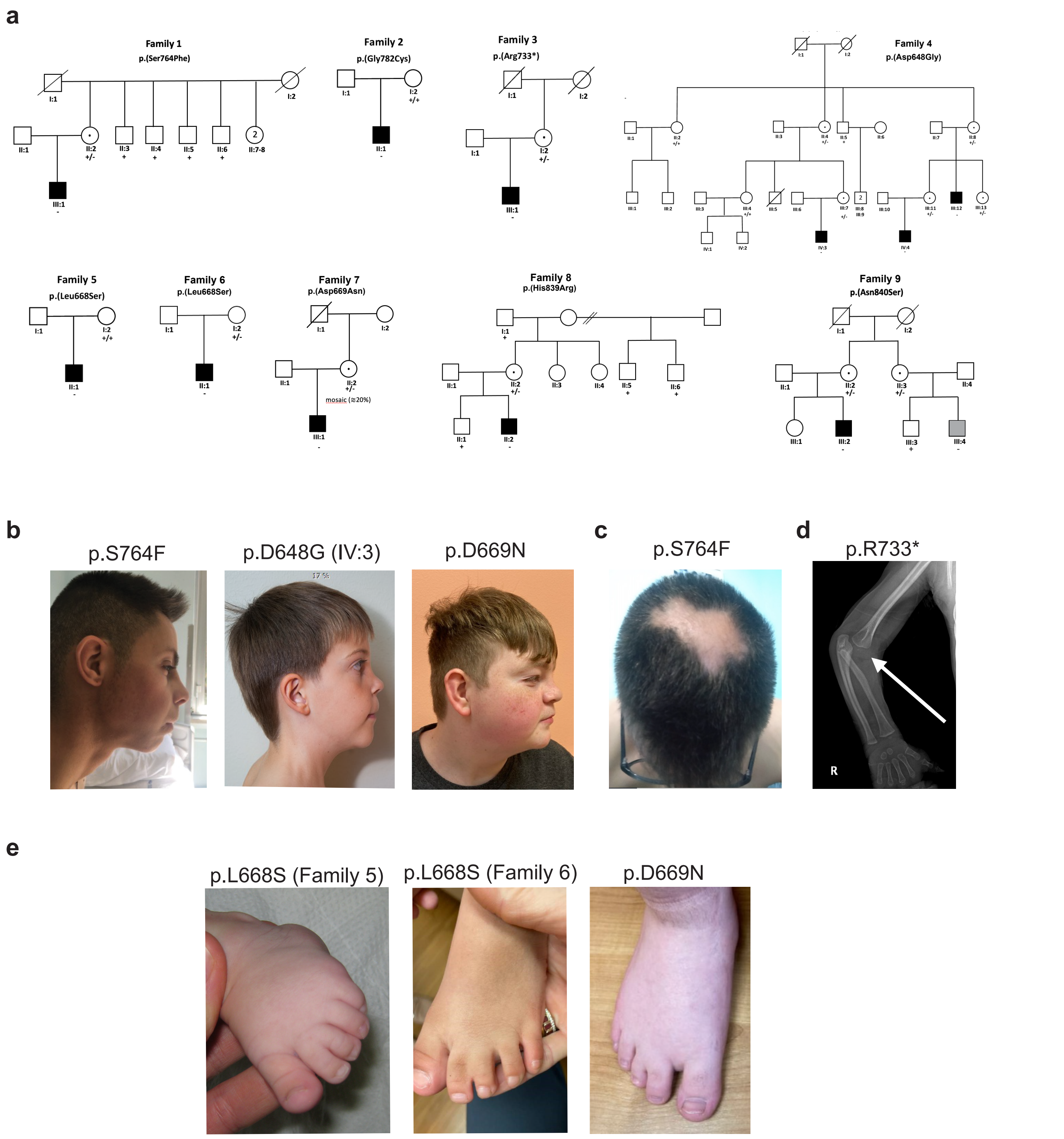

Figure S1: Pedigrees of the families, photos and neuroimaging findings of patients with WDR44 variants (a) Pedigrees of families 1-9. In the pedigrees, squares represent males, circles females, black shaded symbols denote affected patients harboring hemizygous WDR44 variants. Plus (+) and minus (−) signs indicate absence or the presence of the WDR44 variants ([+/+] female wild-type, [+/−] female heterozygote, [+] male wild type, and [−] male hemizygote for the WDR44 variant). The dot indicates the carrier status of females. Note that information on III:4 of family 9 is limited. He is reported to have mild speech delay, febrile seizures, and attention-deficit/hyperactivity disorder (ADHD) (shown in grey shading). (b) Lateral view of patient III:1 of family 1 (p.S764F), IV:3 of family 4 (p.D648G) and III:1 of family 7 (p.D669N) showing midface hypoplasia and retrognathia. (c) Photos of subject III:1 of family 1 showing an area of alopecia on the scalp. (d) Elbow X-ray of subject III:1 of family 3 reveals congenital radial and ulnar heads dislocation (white arrow). (e) Photo of subject II:1 of family 5, II:1 of family 6 and III:1 of family 7 showing II-III toe syndactyly.

a

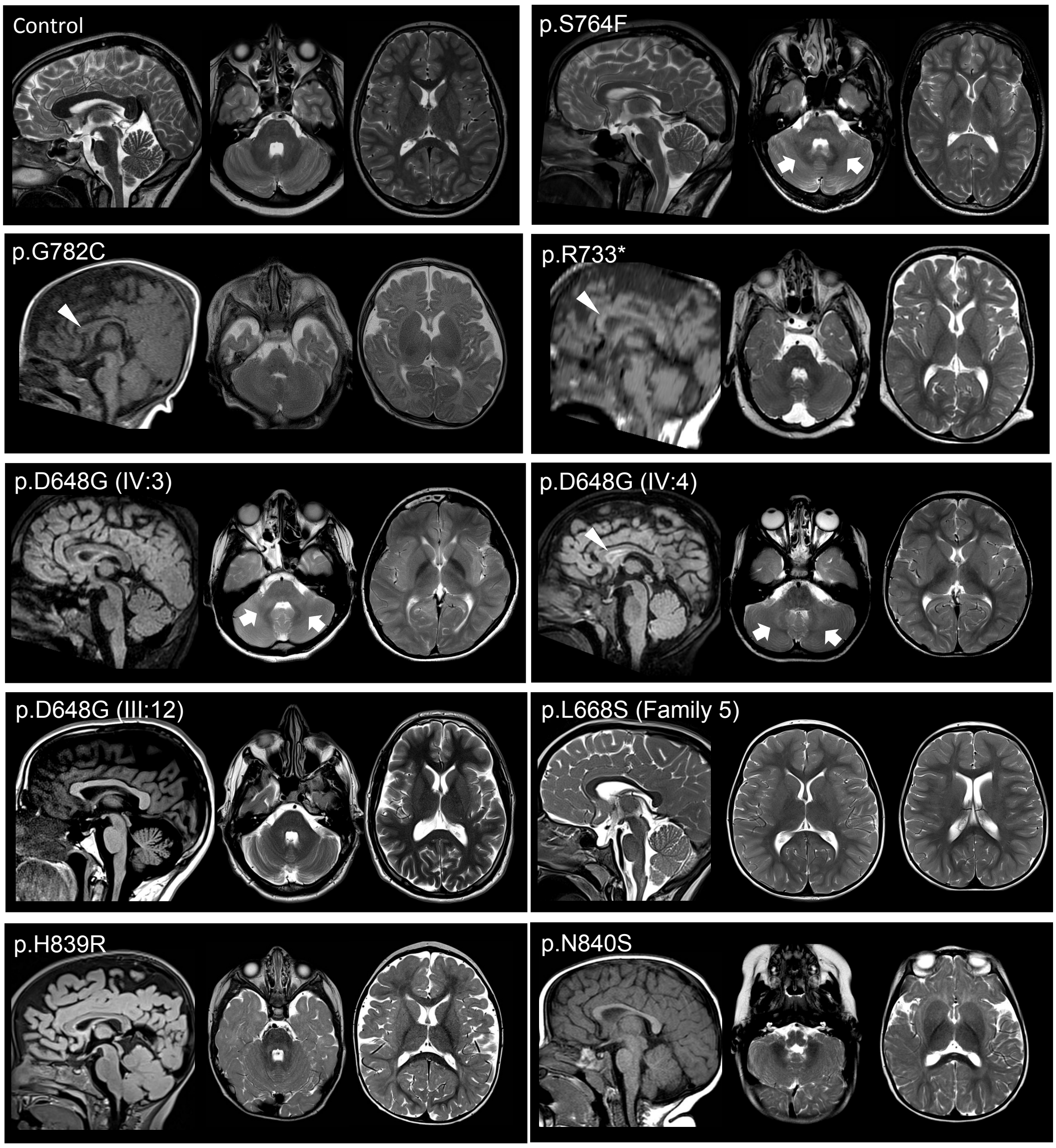

b

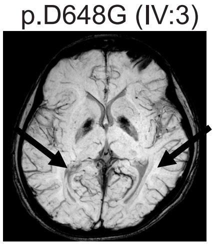

Figure S2: Neuroimaging findings of patients with WDR44 variants
(a) Neuroimaging features of the affected subjects with normal control for comparison. Brain MRI with sagittal T1 or T2 weighted images and axial T2 weighted images showing mild enlargement of the subarachnoid spaces with white matter volume reduction and ventricular enlargement. There is simplification of the gyral pattern and faint T2/FLAIR hyperintensity of the periventricular fronto-parietal white matter and cerebellar white matter (thick arrows). Note the mild corpus callosum dysgenesis, with prevalent anterior involvement in two subjects (arrowheads). (b) Axial susceptibility weighted image of another subject (F4 IV:3) at the age of 16 years reveals bilateral focal hypointensity of the globi pallidi (black arrows).

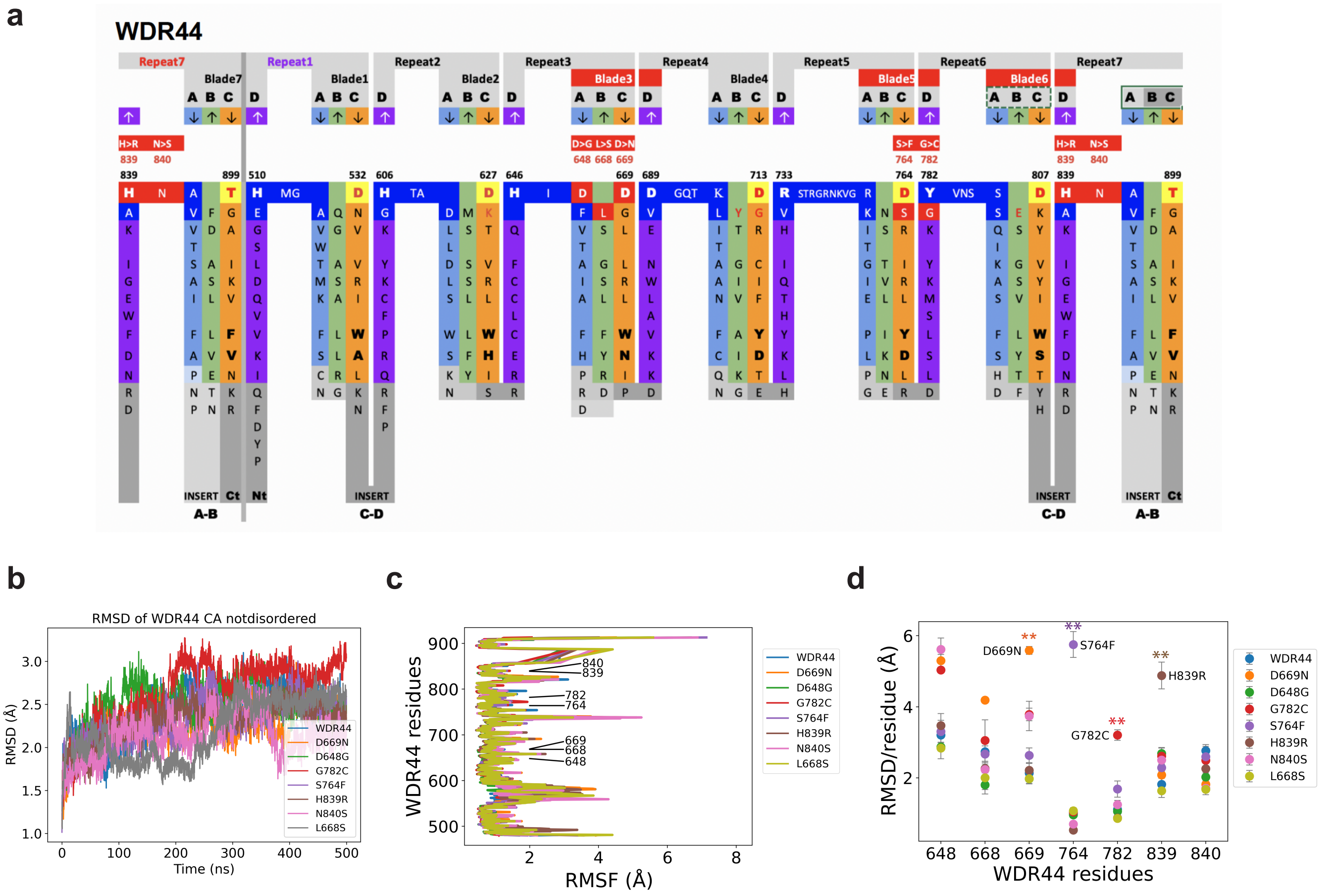

Figure S3: MD simulation and predicted protein structure analysis of WDR44 WDR domain and patient variants effects (a) WDR44 WDR proteomap. Proteomap[62] of the WDR44 WDR shows the sequence and topology of the 7-blade ß-propeller fold, highlighting the positions of patient variants (in red). The proteomap was generated from alignments of WD40 propeller domains across proteins, which shows a highly conserved primary, secondary and tertiary structure across domains, as well as across the seven repeats forming the tertiary domain (see for examples alignment of the WDR44 and WDR5 domains https://structure.ncbi.nlm.nih.gov/icn3d/share.html?nCHMsfcxY2LNqxLE6). The seven repeats are composed, each of the D strand followed by the three strands ABC meander, which assemble sequentially in forming ultimately seven 4-stranded ABCD meanders as blades of the WD40 propeller domain. (b) Cα root mean square deviation (Cα RMSD) profiles of the structured WD40 domains, excluding dynamic disordered region 557-593. Although RMSD profiles fluctuate, they all overlap without showing drastic conformational changes (e.g. large-scale unfolding) over a 500ns simulation time period. (c) All atom root mean square fluctuations (RMSF) profiles of simulated WD40 domains showing dynamic disordered and loop regions. All patient variants exhibit relatively low RMSF values, likely due to their occurrence in ordered WD repeat domains. (d) Per-residue RMSD values, showing that patient variants D669N, S764F, G782C, and H839R have high RMSD values relative to all other simulations. These high RMSD values correspond to local conformational changes as displayed in Figure 2h. Error bars are 95% CI. ** p<0.005 for a one-sided t-test with null hypothesis: the average RMSD/residue of the variant residue is not greater than the average RMSD/residue of the wild-type residue in all other systems. See Methods for calculation details. Source data and all p values are provided as a Source Data file.

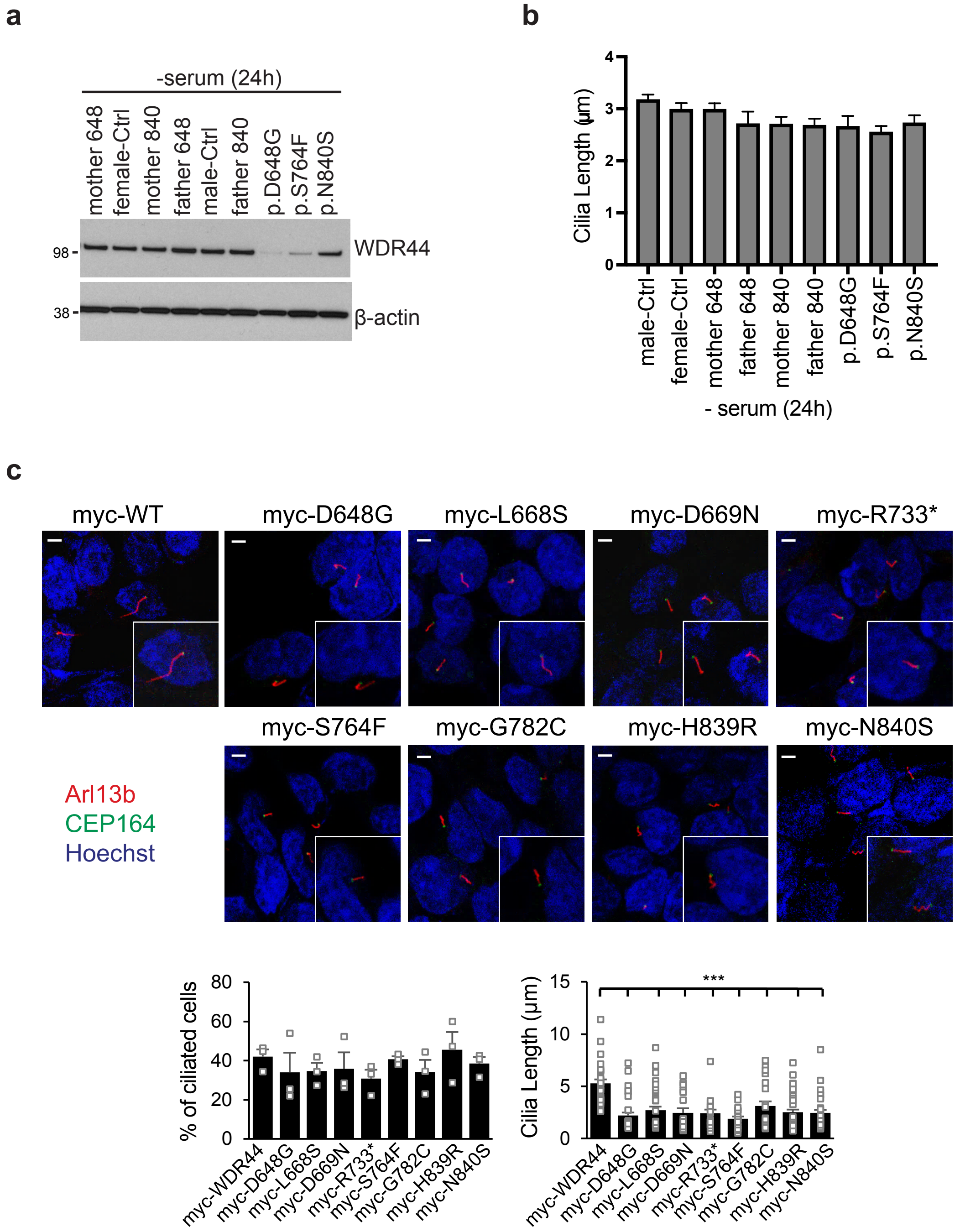

Figure S4: Effect of WDR44 variants on ciliogenesis
(a) Immunoblotting analysis of WDR44 and ß-actin from lysates of control and patient fibroblast grown in high ciliation conditions (starved 24h)(-serum). (b) Quantification of cilia length in fibroblast starved (-serum) for 24h followed by staining with anti-Arl13b, anti-CP110, and anti-CEP164 antibodies. (c) Analysis of ciliation in 293T cells 48 h after transfection of Myc-WDR44 wild-type or variants. IFM images (top) show cilia immunostained with anti-Arl13b and MC with anti-CEP164 antibodies. Nuclei were visualized using Hoechst 33342. Scale bars, 2 µm. Plots show ciliation (left below) and cilia length (right below) quantification in these cells. P = 0.0004 (G782C), <0.0001 (D648G, L668S, D669N, R733*, S764F, H839R, N840S). Mean ± s.e.m. from three independent experiments. Unpaired two-tailed t-test; ***P<0.001. Source data are provided as a Source Data file.

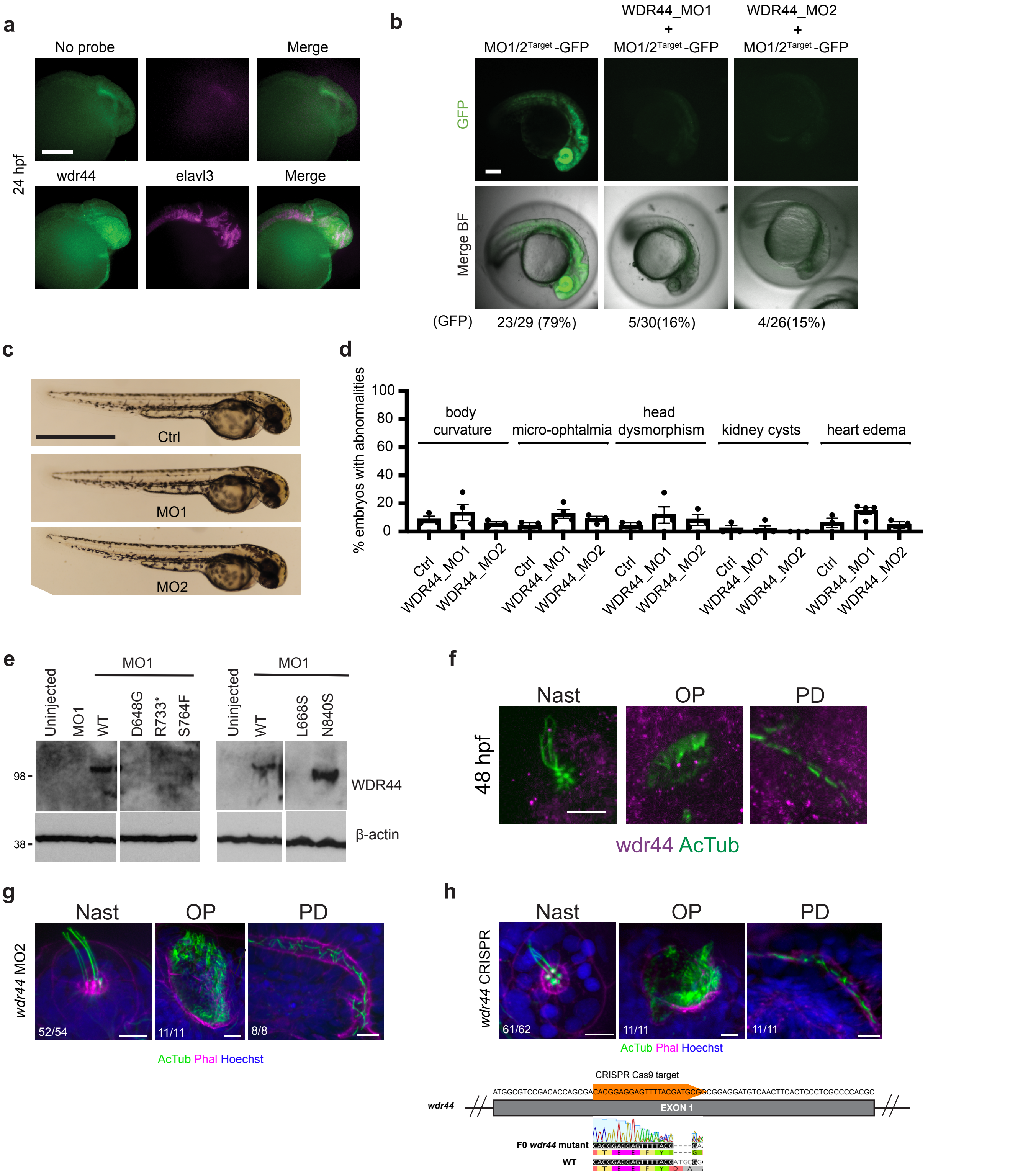

Figure S5: WDR44 zebrafish expression and morpholino knockdown

(a) In-situ HCR analysis of wdr44 mRNA expression (green) in 1dpf embryos. elavl3 mRNA was used as a positive control (purple). No probes were added to the in-situ HCR protocol in our control experiment for non-specific amplification (no probe, upper panels). Scale bars, 200µm (b) wdr44 morpholinos (MO1 and MO2) knockdown efficiency was analyzed by co-injecting the wdr44 morpholinos and pCS2-MO1/2Target-GFP reporter in zebrafish for 24h. Loss of GFP signal represented in IFM images. Scale bars, 200µm. (c, d) Characterization of effects of wdr44 MO1 and MO2 on zebrafish development at 48hpf. (c) BF images. Scale bars, 1mm. Quantification (d) of developmental defects, body curvature, micro-ophtalmia, microcephaly, hydrocephalus, and heart edema. (e) Immunoblotting analysis of wdr44 morphants expressing human WDR44 wild-type or variants at 24hpf. (f) In situ HCR analysis show expression of wdr44 mRNA in ciliated organs (neuromast, Nast; olfactory placode, OP and pronephric duct, PD) from whole embryos fixed and stained with the anti-AcTub antibody at 48hpf. (g) Representative IFM images from 3 dpf embryos injected with wdr44 MO2 stained with the anti-AcTub antibody, Phalloidin, and Hoechst. (h) Representative IFM images (top panels) from 3 dpf embryos injected wdr44 CRISPR gRNA and stained as in (g). (bottom panel) Schematic of CRISPR/Cas9 gRNA targeting exon 1 and Sanger sequencing showing 4 bp deletion. 10 or more embryos were analyzed from three independent experiments (c,d, f,g,h). Scale bars, 10µm. Source data are provided as a Source Data file.

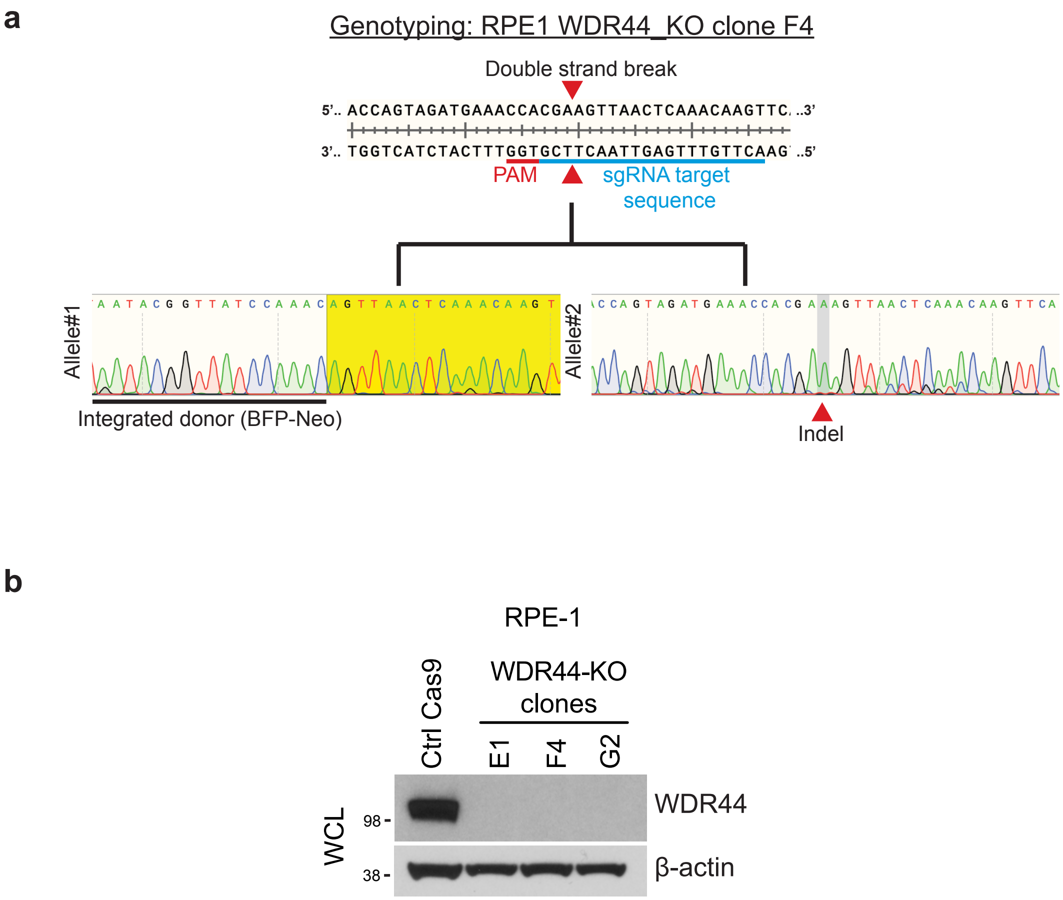

Figure S6: Validation of RPE1 WDR44 KO cell lines

(a) The schematic shows the sgRNA target site. Modified WDR44 alleles of RPE1 WDR44 KO F4 cells were confirmed by genomic DNA Sanger sequencing (below). (b) Immunoblotting analysis of WDR44 and ß-actin from the lysates of RPE-1 control Cas9 and WDR44 knockout (KO) clones.

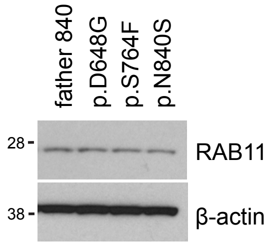

Figure S7: RAB11 expression in WDR44 control and patient variant cells
Immunoblot as described in Figure 2a probed with RAB11 and actin antibodies.

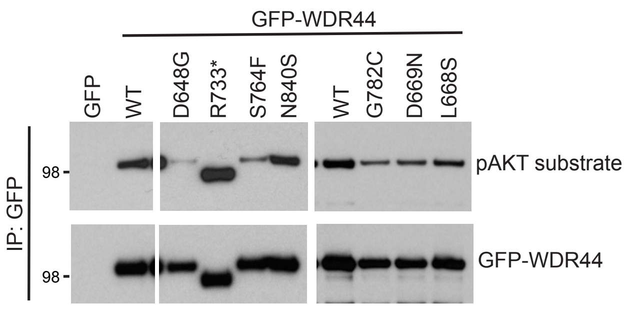

Figure S8: WDR44 variants have unaffected pAKT
Immunoprecipitation of transiently expressed GFP-WDR44 wild-type or variants from 293T cells for 48h. Immunoblots were probed with pAkt-substrate and GFP antibodies.

Table S1. Genetic and phenotypic features of subjects with WDR44 variants

**Family**	**Family 1**	**Family 2**	**Family 3**	**Family 4**			**Family 5**	**Family 6**	**Family 7**	**Family 8**	**Family 9**
**Subject**	III:1	II:1	III:1	IV:4 (ID 370963 - DECIPHER)	IV:3	III:12	II:1	II:1	III:1	III:2	III:2
**WDR44 variant (NM_019045.5)**	c.2291C>T p.(Ser764Phe)	c.2344G>T p.(Gly782Cys)	c.2197C>T p.(Arg733*)	c.1943A>G p.(Asp648Gly)			c.2003T>C p.(Leu668Ser)	c.2003T>C p.(Leu668Ser)	c.2005G>A p.(Asp669Asn)	c.2516A>G p.(His839Arg)	c.2519A>G p.(Asn840Ser)
**Tesing method**	Trio ES	Trio ES	Trio ES	Trio ES	Sanger	ES	Trio ES	Trio ES	ES	Trio ES	Trio ES
**Inheritance**	Maternal	*De novo*	Maternal	Maternal			*De novo*	Maternal	Maternal mosaic (≊20%)	Maternal	Maternal
**Ethnicity**	Italian	South Korea	Russian	Danish			Polish	Hispanic	Caucasian, Native American, Spanish	European	Caucasian
**Sex, age**	M, 21y4m	M, 5y,10m	M, 3y4m	M, 5y7m	M, 15y9m	M, 30y	M, 5y	M, 4y4m	M, 16y9m	M, 3y8m	M, 5y3m
**Pregnancy / neonatal complication**	IUGR, SGA	Late preterm (35weeks)	IUGR, SGA	-	-	-	Late preterm (35weeks), IUGR, SGA	-	Respiratory distress due to TAPVR	IUGR, cystic hygroma	-
**Neurological system**											
DD/ID Behavior abnormalities	*Yes - mild*	*Yes - moderate*	*Yes - moderate*	*Yes - mild*	*Learning disability*	*Learning disability*	*Learning disability*	*Yes - mild*	*Yes - mild*	*Yes - mild*	*Yes - moderate, autism*
Brain MRI findings	Mild simplified gyral pattern, faint T2 hyperintensity of CC, corticospinal tracts, centrum semiovale, cerebellar WM	CC hypoplasia, enlarged CSF spaces, mild WM volume loss, mild ventriculomegaly	Mild simplified gyral pattern, CC hypoplasia, enlarged CSF spaces, moderate WM volume loss, faint T2 hyperintensity of CC, centrum semiovale	Mild simplified gyral pattern, anterior CC hypoplasia, faint T2 hyperintensity of CC, centrum semiovale, cerebellar WM	Mild simplified gyral pattern, faint T2 hyperintensity of CC, centrum semiovale, cerebellar WM	Enlarged CSF space, mild WM volume loss, mild ventriculomegaly	T2 hyperintensity of cerebellar WM	NA	Normal brain findings. Abnormal pituitary gland	Enlarged CSF space, mild WM volume loss, mild ventriculomegaly	Enlarged CSF space, mild WM volume loss, mild ventriculomegaly
Seizure	+ (febrile seizure)	-	-	-	-	-	-	-	-	+	+
Neurological findings	*Hypotonia*	*Hypotonia*	*Hypotonia*	*Hypotonia*	-	-	*Hypotonia*	-	*Hypotonia*	*Hypotonia*	*Hypotonia*
OFC (SD)	*-2.42*	-0.34	NA	*-2.9*	-1.0	NA	-3.3	-1.62	-4.3	-0.97	-1.22

**Dysmorphism**											
Head & face	*High frontal hairline, midface hypoplasia, mild retrognathia,* localized scalp alopecia	*High frontal hairline,micro-retrognathia*	*Sparse hair and high frontal hairline, micro-retrognathia*	*Brachycephaly, retrognathia*	-	-	*High frontal hairline*	*Brachycephaly*	*Bracbycephaly, midface hypoplasia*	*High frontal hairline,* broad forehead, pointed chin	-
Periorbital region	*Upslanting palpebral fissures*, arched eyebrows, infraorbital creases	*Hypertelorism, upslanting palpebral fissures,* arched and thick eyebrows	*Hypertelorism,* thick eyebrows	*Upslanting palpebral fissure,* mild infraorbital crease	*Upslanting palpebral fissure,* infraorbital creases	-	*Upslanting palpebral fissures,* infraorbital creases	*Upslanting palpebral fissures*	*Upslanting palpebral fissure*, epicanthal fold	*Upslanting palpebral fissure,* deep set eyes, broad eyebrows, *long lashes,* infraorbital creases	-
Nose & philtrum	*Long and smooth philtrum*	-	*Long and smooth philtrum*	*Long and smooth philtrum*	*Long and smooth philtrum*	*Long and smooth philtrum*	*Smooth philtrum*	-	*Smooth philtrum*	*Smooth philtrum*	-
Lips & mouth	*Thin upper lip vermilion,high palate, dental crowding*	*High palate, thin upper lip*	*Thin upper vermilion lip, dental crowding*	*Thin upper lip vermilion*	*Thin upper lip vermilion*	*Thin upper lip vermilion*	*Thin upper lip vermilion, high palate*	-	*Thin upper lip vermilion*	*Thin upper lip vermilion*	-
Ears	-	-	Posteriorly rotated ears	-	R ear low set and posteriorly rotated	-	-	Protruding, abnormal helix	-	-	-
Neck & chest	Mild pterygium colli, mild low set R nipple	-	Pterygium colli	-	Pterygium colli, slightly low placed nipples	-	Pterygium colli	-	-	-	-
**Musculoskeletal system**											
Upper limbs	Brachydactyly* (6.9 cm at 21 y ,< 3rd centile)	-	Brachydactyly*, congenital forearms dislocation, ulnar deviation of fingers	Brachydactyly* (4.1 cm at 6y, ,< 3rd centile)	Brachydactyly*(7.2 cm at 17y, <3rd centile), camptodactyly of V fingers	-	*IV-V fingers brachydactyly, fifth finger clinodactyly*	NA	Brachydactyly*(6.8. cm at 16y, <3rd centile	-	-
Spine	*Scoliosis, kyphosis,* L4-L5 schisis	-	*Scoliosis, vertebral bones malformations*	-	*Scoliosis*	-	-	NA	-	-	-
Lower limbs	*Congenital hip dislocation,* (L)	*Congenital hip*	*Congenital hip*	-	Pes planus	-	*Bilateral II-III toes syndactyly*	*Bilateral II-III toes syndactyly*	*Bilateral II-III toes syndactyly*	*Ankle weakness (wears AFOs)*	-

	knee dislocation at birth, *II-V toes brachydactyly, pes planus, talipes equinovarus,* pes planus	*dislocation (L), talipes equinovarus (L)*	*dislocation, talipes equinovarus, II-III toes syndactyly*								
Others	Mild joint hypermobility, hyper CPK (746U/L)	Joint hypermobility	Joint hypermobility, hyper CPK (600-800U/L)	Joint hypermobility, pectus excavatum	Joint hypermobility	Joint hypermobility	-	-	Joint hypermobility	Joint hypermobility	-
**Nephrological system**	*Cysts of right kidney, nephrolithiasis*	-	*Nephritis, CKD2*	-	-	-	*Kidney hypoplasia resulting in CKD2*	NA	-	-	NA
**Urogenital system**	*Cryptorchidism*	*Cryptorchidism*	*Cryptorchidism*	-	-	-	*Cryptorchidism (L)*	-	-	*Cryptorchidism*	-
**Cardiac system**	-	*ASD, mesocardia, bilateral superior vena cava flow*	*ASD*	*Small ASD*	-	-	-	NA	Total anomalous pulmonary venous return	-	-
**Endocrinological system**	*Subclinical hypothyroidism (on L-thyroxine), mild hyperparathyroidism*	-	*Congenital hypothyroidism (ultrasound findings of thyroid hypoplasia)*	-	-	-	-	NA	*Subclinical hypothyroidism, delayed puberty*	-	-
**Hematological system**	Low PLT 115 (× 109/L), WBC 4.39 (× 10^9/L)	-	Low PLT (56-90× 10^9/L); low IgG, IgG, IgA	Intermittent low PLT ((93-144 10^9/L)	-	Low PLT ((103 x 10^9/L)	-	NA	Low WBC (3.8 x 10^9/L, lymphocytes 764)	Low PLT (123 x 10^9/L)	-
**Skin**	*Mildly hyperelastic skin*, Arterovenous fistula of the left arm, bilateral single palmar crease	*Hyperelastic skin, bilateral inguinal hernia, diaphragmatic hernia,* bilateral single palmar crease	*Hyperelastic skin*	Single palmar crease		-	Bilateral single palmar crease, fetal fingertip pads on hands	Interrupted single palmar crease	-	*Mildly hyperelastic skin,* single palmar crease	-
**GI system**	Cholelitiasis	-	-	Gastric ulcers	-	-	-	-	-	-	-
**Other investigations**											

**Microarray (GRCh37/hg 19)**	6q12(66158720 -66369429)x3, 6q2 (165562275- 165819397)x3, both maternally inherited (benign)	19p12(20598 541- 20716336)x1 (benign)	NA	NA	Normal	NA	Normal	4p15.32p15.31 x3 (paternally inherited; benign)	Normal	Normal	8p22(15413434_ 15542785)x1 16p13.11(159984 95_16135150)x1, both maternally inherited (benign)
**Fragile X testing**	Normal	Normal	NA	Normal	Normal	NA	NA	Normal	NA	NA	NA
**Metabolic work-up**	Normal	Normal	NA	NA	NA	NA	7- dehydrocholeste rol normal	NA	NA	Normal	NA

Legend: ASD, atrium septum defect, DD, developmental delay; CC, corpus callosum; CKD2, chronic kidney disease stage 2; CSF, cerebrospinal fluid; CPK, creatine phosphokinase; ES, exome sequencing; GI gastrointestinal system; ID, intellectual disability; IUGR, intrauterine growth retardation; OFC, occipital circumference; L, left; M, male; m, month; NA, not available; SGA, small for gestational age; R, right; SD, standard deviation; TAPVR total anomalous pulmonary venous return; y, year; WBC, white blood cell counts; WM, white matter.

Normal reference for WBC 4.5 to 11.0 × 109/L; PLT, 150 to 400 × 109/L, CPK 22-198U/L.

*According to the lengh of the third finger for specific patient age

Features previously described in ciliopathies are displayed in italics.

Table S2: Allele frequency of *WDR44* variants and predictive analyses performed with multiple *in-silico* tools.

Family No.	Nucleotide alteration[a]	Coding Sequence alteration[b]	Amino acid alteration	AF gnomAD (v3.1.2)	PhyloP 100way[c]	GERP++[l]	dN/dS[e]	Polyphen[f]	Provean[g]	MUpro[h] ΔΔG	SIFT[i]	Mutation Taster[l]	CADD[m]	REVEL[n]	ACMG score system	ACMG classification
1	chrX:118442587C>T	c.2291C>T	p.(Ser764Phe)	-	7.439	5.67	0.3 (intolerant)	1.00 (D)	- 5.790 (D)	- 0.426 (DS)	0.001 (D)	DC	29.3	0.93 (D)	PS3_sup PM2_sup PP1_mod PP3_mod PP4_sup	LP (7)
2	chrX:118442640G>T	c.2344G>T	p.(Gly782Cys)	-	7.7	5.96	0.17 (highly intolerant)	1.00 (D)	-8.56 (D)	- 1.095 (DS)	0.03 (D)	DC	29.9	0.87 (D)	PS2_strong PS3_sup PM2_sup PP3_mod PP4_sup	LP (9)
3	chrX:118442274C>T	c.2197C>T	p.(Arg733*)	-	4.88	5.43	na	na	na	na	D	DC	39	0.67 (D)	PS3_sup PM2_sup PP3_mod PP4_sup	VUS (5)
4	chrX:118436793A>G	c.1943A>G	p.(Asp648Gly)	-	9.188	5.96	0.52 (intolerant)	1.00 (D)	-6.585 (D)	-1.825 (DS)	0.08 (D)	DC	32	0.85 (D)	PS3_sup PM2_sup PP1_strong PP3_mod PP4_sup	LP(9)
5-6	chrX:118441396T>C	c.2003T>C	p.(Leu668Ser)	-	7.969	5.61	0.2 (intolerant)	1.000 (D)	-5.463 (D)	-2.055 (DS)	0.02 (D)	DC	27.4	0.63 (borderline D)	PS2_strong PS3_sup PM1_mod PM2_sup PP3_sup PP4_sup	P (10)
7	chrX:118441398G>A	c.2005G>A	p.(Asp669Asn)	-	9.94	5.48	0.29 (intolerant)	1.000 (D)	-4.747 (D)	-0.891 (DS)	0.00 (D)	DC	29.5	0.86 (D)	PS3_sup PM1_mod PM2_sup	LP(7)

															PP3_mod PP4_sup	
8	ChrX:118444363A>G	c.2516A>G	p.(His839Arg)	-	7.743	5.34	0.76 (neutral)	1.000 (D)	-6.969 (D)	-0.947 (DS)	0.004 (D)	DC	24.4	0.88 (D)	PS3_sup PM2_sup PP1_mod PP4_sup	VUS(5)
9	chrX:118444366A>G	c.2519A>G	p.(Asn840Ser)	0.0000178 (2/112.163 Het)	7.743	5.34	0.76 (neutral)	0.589 (Possibly D)	-2.767	-0.903	0.049 (D/T)	DC	22.4	0.17 (T)	PS3_sup PM2_sup PP1_sup PP4_sup	VUS(4)

Legend: AF, allele frequency; bg, background of possible missense and synonymous variants based on the codon table; D, deleterious; DC, disease causing; dN, $missense_{obs}/missense_{bg}$. dS, $synonymous_{obs}/synonymous_{bg}$; DS decrease stability; het heterozygous; mod, moderate; LP, likely pathogenic; NA, not applicable; P, pathogenic; sup, supporting.

[a]Positions refer to GRCh38/hg38. [b]Positions refer to GenBank: NM_019045.5. [c]phyloP100 way score: **PhyloP100way** scores are based on multiple alignments of 99 vertebrate genome sequences to the human genome. The greater the score, the more conserved the site. [d]**GERP++** score: DNA conservation score ranges from -12.3 to 6.17, with 6.17 being the most conserved. [e]**dN/dS (MetaDome)** score: it depicts a missense over synonymous ratio (also known as Ka/Ks, or dN/dS) based on the gnomAD dataset with a sliding window of 21 residues over the entirety of the protein of interest (e.g., calculated for 10 residues left and right the residue of interest). [f]**PolyPhen2 HDIV** score: variants with scores between 0.85 and 1.0 are predicted to be damaging (D) with high confidence. [g] **PROVEAN** (Protein Variation Effect Analyzer) predicts the impact of single or multiple amino acid substitutions, and in-frame insertions and deletions based on alignment-based scores derived from pairwise sequence alignments between the query sequence and each of the related sequences at the protein level (range -14 to +14; score ≤ -2.5 is considered deleterious). [h] **MUpro** (Prediction of Protein Stability Changes for Single-Site Mutations from Sequences) predicts protein stability changes for single amino acid mutations using support vector machine (SVM) model. Variants with scores less than 0 are predicted to decrease the protein stability. [i]**SIFT**: function prediction tool based on protein sequence conservation among homologs. Variants with scores between 0 and 0.05 are considered deleterious / to affect protein function. [l]**MutationTaster** predicts through a Bayes classifier the probability for the alteration to be either a disease mutation or a harmless polymorphism. [m]**CADD**v1.4 scores range from 1 to 99, with a higher score indicating greater deleteriousness. [n]**REVEL:** is an ensemble method for predicting the pathogenicity of missense variants based on a combination of scores from 13 individual tools: MutPred, FATHMM v2.3, VEST 3.0, PolyPhen-2, SIFT, PROVEAN, MutationAssessor, MutationTaster, LRT, GERP++, SiPhy, phyloP, and phastCons.The REVEL score for an individual missense variant can range from 0 to 1, with higher scores reflecting greater likelihood that the variant is disease-causing. ACMG score system from Tavtigian et al [63]. Final scoring is indicated in brackets. PP3 calculated according to the CADD score following the updated guidelines[64].

Table S3. (A) Breakdown of high quality autosomal SNVs in family 2 proband demonstrating Mendelian inheritance pattern.

Inheritance pattern*		# of SNVs	%
Expected patterns in a trio	AA x AB = AB	3466	11.36%
	AB x AA = AB	3369	11.04%
	AA x BB = AB	1269	4.16%
	BB x AA = AB	1429	4.68%
	AB x BB = AB	1225	4.01%
	BB x AB = AB	1361	4.46%
	AB x AB = AB	3360	11.01%
	AB x AB = BB	1528	5.01%
	AB x BB = BB	1972	6.46%
	BB x AB = BB	1643	5.38%
	BB x BB = BB	9875	32.36%
	Total	30497	99.94%
Unexpected patterns in a trio	AA x AA = AB	5	
	AA x AB = BB	3	
	AA x BB = BB	6	
	AB x AA = BB	1	
	BB x AA = BB	4	
	Total	19**	0.06%

*. Denotes genotypes of (mother) x (father) = (proband). "A" and "B" refer to reference allele and non-reference allele, respectively.
**. Due to true *de novo* calls, low covered calls or spurious calls.

(B) Segregation analysis of three ultrarare variants identified by exome sequencing in family 5. X denotes presence of the genetic variant.

**Variant – genomic position hg38**	**Proband**	**Mother**	**Father**
WDR26 1:224411450-C>T	x		x
PAXBP1 21:032769808-A>T	x	x	
CLDN5 22:019523698-C>T	x	x	

Table S4: Primers used in study

Primer	Sequence
WDR44-pCMV6-F	5'-AGATCTGCCGCCGCGATCGCCATGGCGTCGG-3'
WDR44-pCMV6-R	5'-GAGCGGCCGCGTACGCGTAGATACATTTTTTC-3'
WDR44 D648G-R	5'-AGTGACAAAACCTATATGTTGAAAACAGCAAAGGC-3'
WDR44 D648G-F	5'-CAACATATAGGTTTTGTCACTGCCATAGCTTTTCATC-3'
WDR44 L668S-R	5'-CTTTCCATCCGAAGACCCACTTAGAAAATACCTGTCATCT CTTG-3'
WDR44 L668S-F	5'-AGTGGGTCTTCGGATGGAAAGCTCCGCCTTTGG-3'
WDR44 D669N-R	5'-GAGCTTTCCATTCAAAGACCCACTTAGAAAATACCTGTCA TCTCT TG-3'
WDR44 D669N-F	5'-GGGTCTTTGAATGGAAAGCTCCGCCTTTGG-3'
WDR44 R733*-R	5'-TCTAGTAGATCAGACATGTATTTGTGTATGGTATTTCAAAT GC-3'
WDR44 R733*-F	5'-ATACATGTCTGATCTACTAGAGGGCGCAACAAG-3'
WDR44 R733*-R2	5'-CGGCCGCGTTCGCGTGACATGTATTTGTG-3'
WDR44 R733*-F3	5'-GTCACGCGAACGCGGCCGCTCGAGCAG-3'
WDR44 R733*-R3	5'-AGTAGATCAAACCTTATCGTCGTCATCCTTG-3'
WDR44 R733*-F4	5'-GATAAGGTTTGATCTACTAGAGGGCGCAAC-3'
WDR44 R733*-R4	5'-AGTAGATCAAACCTTATCGTCGTCATCCTTGTAATCCAGG ATATC-3'
WDR44 S764F-R	5'-TCTGATTCTGAAGTCATTTGAGGTTACCAATATCTTATTTT CTC-3'
WDR44 S764F-F	5'-TCAAATGACTTCAGAATCAGACTATATGATTTGAGAGATT TG-3'
WDR44 G782C-R	5'-ATTGACGTAACACTTATACTTCATGGATAGTGACAAATCT C-3'
WDR44 G782C-F	5'-GAAGTATAAGTGTTACGTCAATAGCAGCAGCCAGA-3'
WDR44 H839R-R	5'-AACTGCATTGCGGGCTTTAATACCTTCCCAGAAGTC-3'
WDR44 H839R-F	5'-ATTAAAGCCCGCAATGCAGTTGTTACATCAGCCATC-3'
WDR44 N840S-R	5'-AACAACTGCACTGTGGGCTTTAATACCTTCCCAG-3'
WDR44 N840S-F	5'-AAAGCCCACAGTGCAGTTGTTACATCAGCCATC-3'
NH_2-HA-R	5'-GCGGCCGCGTACGCGTATTAAGCGTAATCTGGAACAT CGTATGGG TACTTCTCTTCATCATCTGACTGTGC-3'
COOH-MYC-F	5'-AGATCTGCCGCCGCGATCGCCATGTTACAGTCTCAGCCA ACAGAT ACTGATGGTG-3'
COOH-MYC-R	5'-CAGATCCTCTTCTGAGATGAGTTTCTGCTCAGATACATTT TTTCTTT TATTAACAAACACTTTGATTG-3'
COOH-MYC-R2	5'-GCGGCCGCGTACGCGTATTACAGATCCTCTTCTGAGATG AGTTTC-3'
COOH-GST-F	5'-AGATCTGCCGCCGCGATCGCCATGATGCCATACACAAGAC CAG-3'
COOH-GST-R	5'-GAGTCCGGAAGATACATTTTTTCTTTTATTAACAAACACTT TG-3'

`

COOH-GST-F2	5'-GTTAATAAAAGAAAAAATGTATCTTCCGGACTCAGATCTC GAGCC TC-3'
COOH-GST-R2	5'-GCGGCCGCGTACGCGTATTATTTTGGAGG-3'
pDONR221 WDR44-D648G-F	5'-CAACATATAGGTTTTGTCACTG-3'
pDONR221 WDR44-D648G-R	5'-AAAACAGCAAAGGCATTC-3'
pDONR221 WDR44-L668S-F	5'-TCTTCGGATGGAAAGCTCC-3'
pDONR221 WDR44-L668S-R	5'-CCCACTTAGAAAATACCTGT-3'
pDONR221 WDR44-D669N-F	5'-TGGGTCTTTGAATGGAAAGCTC-3'
pDONR221 WDR44-D669N-R	5'-CTTAGAAAATACCTGTCATCTC-3'
pDONR221 WDR44-S764F-F	5'-TCAAATGACTTCAGAATCAGAC-3'
pDONR221 WDR44-S764F-R	5'-GGTTACCAATATCTTATTTTCTC-3'
pDONR221 WDR44-G782C-F	5'-GAAGTATAAGTGTTACGTCAATAGC-3'
pDONR221 WDR44-G782C-R	5'-ATGGATAGTGACAAATCTC-3'
pDONR221 WDR44-H839R-F	5'-ATTAAAGCCCGCAATGCAGTTG-3'
pDONR221 WDR44-H839R-R	5'-ACCTTCCCAGAAGTCATTAC-3'
pDONR221 WDR44-N840S-F	5'-AAAGCCCACAGTGCAGTTGTT-3'
pDONR221 WDR44-N840S-R	5'-AATACCTTCCCAGAAGTC-3'
pDONR221 WDR44-R733*-F	5'-AATACATGTCTGATCTACTAGAG-3'
pDONR221 WDR44-R733*-R	5'-TGTGTATGGTATTTCAAATG-3'

`
