# Supplementary material for: Variants in the WDR44 WD40-repeat domain cause a spectrum of ciliopathy by impairing ciliogenesis initiation
Source: Nat Commun. 2024 Jan 8;15:365. doi: 10.1038/s41467-023-44611-2 (PMC10774338; doi:10.1038/s41467-023-44611-2)
Supplement: Supplementary file 3 — Description of Additional Supplementary Files [file 41467_2023_44611_MOESM3_ESM.pdf]

Description of Additional Supplementary Files

File Name: Supplementary Data 1
Description: Rare variants filtered from exome data analysis of subject III:1 in family 1
