# Supplementary material for: Variants in the WDR44 WD40-repeat domain cause a spectrum of ciliopathy by impairing ciliogenesis initiation
Source: Nat Commun. 2024 Jan 8;15:365. doi: 10.1038/s41467-023-44611-2 (PMC10774338; doi:10.1038/s41467-023-44611-2)
Supplement: Supplementary file 5 — Reporting Summary [file 41467_2023_44611_MOESM5_ESM.pdf]

nature portfolio

Corresponding author(s): Chris Westlake

Last updated by author(s): Oct 27 2023

Reporting Summary

Nature Portfolio wishes to improve the reproducibility of the work that we publish. This form provides structure for consistency and transparency in reporting. For further information on Nature Portfolio policies, see our Editorial Policies and the Editorial Policy Checklist.

Please do not complete any field with "not applicable" or n/a. Refer to the help text for what text to use if an item is not relevant to your study.
For final submission: please carefully check your responses for accuracy; you will not be able to make changes later.

Statistics

For all statistical analyses, confirm that the following items are present in the figure legend, table legend, main text, or Methods section.

n/a	Confirmed	
☐	☑	The exact sample size (*n*) for each experimental group/condition, given as a discrete number and unit of measurement
☐	☑	A statement on whether measurements were taken from distinct samples or whether the same sample was measured repeatedly
☐	☑	The statistical test(s) used AND whether they are one- or two-sided<br>*Only common tests should be described solely by name; describe more complex techniques in the Methods section.*
☒	☐	A description of all covariates tested
☐	☑	A description of any assumptions or corrections, such as tests of normality and adjustment for multiple comparisons
☐	☑	A full description of the statistical parameters including central tendency (e.g. means) or other basic estimates (e.g. regression coefficient) AND variation (e.g. standard deviation) or associated estimates of uncertainty (e.g. confidence intervals)
☐	☑	For null hypothesis testing, the test statistic (e.g. *F*, *t*, *r*) with confidence intervals, effect sizes, degrees of freedom and *P* value noted<br>*Give P values as exact values whenever suitable.*
☒	☐	For Bayesian analysis, information on the choice of priors and Markov chain Monte Carlo settings
☒	☐	For hierarchical and complex designs, identification of the appropriate level for tests and full reporting of outcomes
☒	☐	Estimates of effect sizes (e.g. Cohen's *d*, Pearson's *r*), indicating how they were calculated

Our web collection on statistics for biologists contains articles on many of the points above.

Software and code

Policy information about availability of computer code

Data collection	Excel Patients data collected through recruitment as described in methods, Gene Matcher, public database (e.g. DECIPHER) Telethon Undiagnosed Disease Program, European Network of Rare Malformation Syndromes.<br>Brain MRI, Sonogram, Zeiss Axio scan Z1 inverted epifluorescence microscope equipped with a CoolSNAP HQ2 camera or Zeiss LSM710 confocal microscope, Applied Biosystems 7500 Fast Real Time PCR system, CFX96 Bio-Rad thermocycler
Data analysis	GATK Best Practices pipeline, Visual Molecular Dynamics (VMD) mutator, Variants stability analysed by PhyloP100way, GERP++, dN/dS (MetaDome), PolyPhen2, PROVEAN, MUpro, SIFT, MutationTaster, CADD, REVEL tools. Slidebook software and ZEN software (Zeiss) were used for image analysis. GraphPad Prism

Data

Policy information about availability of data

All manuscripts must include a data availability statement. This statement should provide the following information, where applicable:
- Accession codes, unique identifiers, or web links for publicly available datasets
- A description of any restrictions on data availability
- For clinical datasets or third party data, please ensure that the statement adheres to our policy

All data that support the findings in this paper are available within the article and its supplementary information files. Source data are provided in the paper.

Research involving human participants, their data, or biological material

Policy information about studies with human participants or human data. See also policy information about sex, gender (identity/presentation), and sexual orientation and race, ethnicity and racism.

Reporting on sex and gender

Reporting on race, ethnicity, or other socially relevant groupings

We collected information on sex of the patients to establish whether gender influences any aspect of the natural history of the disease that is specifically relevant for X-linked disorder. This has also implications for family to offer risk of recurrence and counselling. We also collected information related to patients ethnicity to understand whether ethnic background may correlate with individual genetic variants or recurrence of the disease in certain populations.

Population characteristics: Age of paients ranges from 3 years and 8 months to 21 years and 4 months.

Recruitment: Patients - cohort of 11 individuals was recruited via an international collaborative network of research sequencing laboratories, GeneMatcher Exchange platform, the European Network of Rare Malformation Syndromes (ERN-ITHACA), public databases such as DECIPHER or personal communication from different institutions

Ethics oversight: The study was approved by the ethics committee of Telethon Institute of Genetics and Medicine (TIGEM), Naples, Italy (number of protocol 81/21) and additional local ethics committees of the participating centers.

Note that full information on the approval of the study protocol must also be provided in the manuscript.

Field-specific reporting

Please select the one below that is the best fit for your research. If you are not sure, read the appropriate sections before making your selection.

☑ Life sciences ☐ Behavioural & social sciences ☐ Ecological, evolutionary & environmental sciences

For a reference copy of the document with all sections, see nature.com/documents/nr-reporting-summary-flat.pdf

Life sciences study design

All studies must disclose on these points even when the disclosure is negative.

Sample size: the number of zebrafish embryo or ciliated organs counted is indicated by n from at least 3 biological replicates. Cell numbers counted for cilia determination are indicated in methods or figure legends. For cell culture experiments, >150 cells were counted to quantify ciliation and >100 cells were counted for anlyzing CP110 loss from at least 3 biological replicates. No predetermined sample-size calculations were done but sample sizes that are used are an accepted sample size for the methods used.

Data exclusions: n/a

Replication: Three or four independent experiments were performed

Randomization: For randomization of zebrafish experiments, each injected group was assigned a random number.

Blinding: Investigators were blinded to the group allocation of zebrafish during the data collection and analysis

Behavioural & social sciences study design

All studies must disclose on these points even when the disclosure is negative.

Study description

Research sample

Sampling strategy

Data collection

Timing

Data exclusions

Non-participation

Randomization

Ecological, evolutionary & environmental sciences study design

All studies must disclose on these points even when the disclosure is negative.

Study description

Research sample

Sampling strategy

Data collection

Timing and spatial scale

Data exclusions

Reproducibility

Randomization

Blinding

Did the study involve field work? ☐ Yes ☐ No

Field work, collection and transport

Field conditions

Location

Access & import/export

Disturbance

Reporting for specific materials, systems and methods

We require information from authors about some types of materials, experimental systems and methods used in many studies. Here, indicate whether each material, system or method listed is relevant to your study. If you are not sure if a list item applies to your research, read the appropriate section before selecting a response.

Materials & experimental systems

n/a	Involved in the study
☐	☑ Antibodies
☐	☑ Eukaryotic cell lines
☒	☐ Palaeontology and archaeology
☐	☑ Animals and other organisms
☐	☑ Clinical data
☒	☐ Dual use research of concern
☒	☐ Plants

Methods

n/a	Involved in the study
☒	☐ ChIP-seq
☒	☐ Flow cytometry
☐	☑ MRI-based neuroimaging

Antibodies

Antibodies used

Antibodies purchased from
Millipore Sigma: acetylated a-tubulin (Actub) (Catalog no. T7451), CP110 (Catalog no. MABT1354), β-Actin-HRP (Catalog no. A3854).
Proteintech: Arl13b (Catalog no. 17711-1- AP), VAPA (Catalog no. 15275-1-AP), LC3 (Catalog no. 14600-1-AP).
SantaCruz: CEP164 (Catalog no. sc-515403), GFP (Catalog no. sc-9996), GAPDH (Catalog no. sc-166574)
Cell Signaling: Akt (pan) (Catalog no. 4691), Phospho Akt substrate (RXRXXS*/T*) 23C8D2 (Catalog no. 10001S), Phospho-AKT S473 (Catalog no. 4060S), β-Actin (Catalog no. 4970), p27 (Catalog no. 3698), Myc (Catalog no. 2276), HA (Catalog no. 3724), GST (Catalog no. 2622). ThermoFisher Scientific: Rab11 (Catalog no. 71- 5300), Alexa Fluor 488 donkey anti-mouse (Catalog no. A-32766), Alexa Fluor 568 donkey anti-rabbit (Catalog no. A10042).
Bethyl: WDR44 (Catalog no. A301-440A).
Labmade: Cep164 (CEP164N: 1-400 aa; NM_014956) is generated in laboratory.
Miltenyi Biotec: GFP-HRP (Catalog no. 130-091-833).
Bio-Rad: hFAB Rhodamine GAPDH antibody (Catalog no. 12004167).
Jackson ImmunoReasearch Lab: Alexa Fluor 647 donkey anti-chicken IgY (Catalog no. 703-605-155)
GE Healthcare: HRP-conjugated donkey anti-rabbit (Catalog no. NA934V).

Validation

Validation - All antibodies that are used in study are validated either by commercial manufacturer or by cited references. We have also confirm the specificity of these antibodies by checking the molecular weight of proteins by WB, knock down of endogenous proteins and by immunostaning.
Laboratory made Cep164
(CEP164N: 1-400 aa; NM_014956) antibody specificity was determined by co-staining with commmercial CEP164 antibody and Knock down of endogenous CEP164

Eukaryotic cell lines

Policy information about cell lines and Sex and Gender in Research

Cell line source(s)	hTERT-RPE-1 from ATCC 293T and hTERT-RPE cells (RPE-1) were purchased from American Tissue Culture Collection. WDR44 knock-out RPE-1 cell line were generated using the homology-independent knock-in system as described in methods. . Human dermal Fibroblasts were isolated from patients skin biopsiesPatient fibroblast and controls (p.N840S, p.S764F, p.D648G, mother 840,father 850, father 648, mother 648, male Ctrl from clinical preparations.WDR44 stable fibroblasts were prepared by lentivirus infection as decribed in methods.
Authentication	RPE)-1 and 293T cells from ATCC
Mycoplasma contamination	negative
Commonly misidentified lines (See ICLAC register)	No commonly misidentifed cell lines used in this study

Palaeontology and Archaeology

Specimen provenance	
Specimen deposition	
Dating methods	

☐ Tick this box to confirm that the raw and calibrated dates are available in the paper or in Supplementary Information.

Ethics oversight	

Note that full information on the approval of the study protocol must also be provided in the manuscript.

Animals and other research organisms

Policy information about studies involving animals; ARRIVE guidelines recommended for reporting animal research, and Sex and Gender in Research

Laboratory animals	AB strain danio rerio
Wild animals	no
Reporting on sex	no
Field-collected samples	no
Ethics oversight	maintained in accordance with the protocols (ASP#20-416) approved by the Animal Care and Use Committee of the National Cancer Institute at Frederick and AAALAC guidelines

Note that full information on the approval of the study protocol must also be provided in the manuscript.

Clinical data

Policy information about clinical studies

All manuscripts should comply with the ICMJE guidelines for publication of clinical research and a completed CONSORT checklist must be included with all submissions.

Clinical trial registration	n/a
Study protocol	in methods
Data collection	in methods
Outcomes	n/a

Dual use research of concern

Policy information about dual use research of concern

Hazards

Could the accidental, deliberate or reckless misuse of agents or technologies generated in the work, or the application of information presented in the manuscript, pose a threat to:

No	Yes	
[x]	[ ]	Public health
[x]	[ ]	National security
[x]	[ ]	Crops and/or livestock
[x]	[ ]	Ecosystems
[x]	[ ]	Any other significant area

Experiments of concern

Does the work involve any of these experiments of concern:

No	Yes	
[x]	[ ]	Demonstrate how to render a vaccine ineffective
[x]	[ ]	Confer resistance to therapeutically useful antibiotics or antiviral agents
[x]	[ ]	Enhance the virulence of a pathogen or render a nonpathogen virulent
[x]	[ ]	Increase transmissibility of a pathogen
[x]	[ ]	Alter the host range of a pathogen
[x]	[ ]	Enable evasion of diagnostic/detection modalities
[x]	[ ]	Enable the weaponization of a biological agent or toxin
[x]	[ ]	Any other potentially harmful combination of experiments and agents

Plants

Seed stocks	
Novel plant genotypes	
Authentication	

ChIP-seq

Data deposition

- [ ] Confirm that both raw and final processed data have been deposited in a public database such as GEO.
- [ ] Confirm that you have deposited or provided access to graph files (e.g. BED files) for the called peaks.

Data access links<br>*May remain private before publication.*	
Files in database submission	
Genome browser session<br>(e.g. UCSC)	

Methodology

Replicates	
Sequencing depth	
Antibodies	
Peak calling parameters	
Data quality	
Software	

Flow Cytometry

Plots

Confirm that:

☐ The axis labels state the marker and fluorochrome used (e.g. CD4-FITC).

☐ The axis scales are clearly visible. Include numbers along axes only for bottom left plot of group (a 'group' is an analysis of identical markers).

☐ All plots are contour plots with outliers or pseudocolor plots.

☐ A numerical value for number of cells or percentage (with statistics) is provided.

Methodology

Sample preparation	
Instrument	
Software	
Cell population abundance	
Gating strategy	

☐ Tick this box to confirm that a figure exemplifying the gating strategy is provided in the Supplementary Information.

Magnetic resonance imaging

Experimental design

Design type	in methods
Design specifications	n/a
Behavioral performance measures	n/a

Imaging type(s)	human brain
Field strength	n/a
Sequence & imaging parameters	in methods
Area of acquisition	in methods

Diffusion MRI ☐ Used ☑ Not used

Preprocessing

Preprocessing software	various labs so not applicable
Normalization	n/a
Normalization template	n/a
Noise and artifact removal	unknown
Volume censoring	unknown

Statistical modeling & inference

Model type and settings	n/a
Effect(s) tested	n/a

Specify type of analysis: ☑ Whole brain ☐ ROI-based ☐ Both

Statistic type for inference

(See Eklund et al. 2016)

n/a

Correction

unknown

Models & analysis

n/a	Involved in the study
x	☐ Functional and/or effective connectivity
x	☐ Graph analysis
x	☐ Multivariate modeling or predictive analysis

Functional and/or effective connectivity

n/a

Graph analysis

n/a

Multivariate modeling and predictive analysis

n/a

This checklist template is licensed under a Creative Commons Attribution 4.0 International License, which permits use, sharing, adaptation, distribution and reproduction in any medium or format, as long as you give appropriate credit to the original author(s) and the source, provide a link to the Creative Commons license, and indicate if changes were made. The images or other third party material in this article are included in the article's Creative Commons license, unless indicated otherwise in a credit line to the material. If material is not included in the article's Creative Commons license and your intended use is not permitted by statutory regulation or exceeds the permitted use, you will need to obtain permission directly from the copyright holder. To view a copy of this license, visit http://creativecommons.org/licenses/by/4.0/
